# Short-term effects of vacuum massage on epidermal and dermal thickness and density in burn scars: an experimental study

**DOI:** 10.1186/s41038-016-0052-x

**Published:** 2016-07-08

**Authors:** Jill Meirte, Peter Moortgat, Mieke Anthonissen, Koen Maertens, Cynthia Lafaire, Lieve De Cuyper, Guy Hubens, Ulrike Van Daele

**Affiliations:** 1 OSCARE, Organisation for Burns, Scar After-care and Research, Van Roiestraat 18, 2170 Antwerp, Belgium; 2 Rehabilitation Sciences and Physiotherapy, University of Antwerp, Universiteitsplein 1, 2610 Antwerp, Belgium; 3 KU Leuven, Faculty of Kinesiology and Rehabilitation Sciences, Leuven, Belgium; 4 Department of Clinical and Lifespan Psychology, Vrije Universiteit Brussel, Brussels, Belgium; 5 ZNA Stuivenberg, Burn Center, Antwerp, Belgium; 6 Department of Antwerp Surgical Training Anatomy and Research Centre, University of Antwerp, Antwerp, Belgium

**Keywords:** Burn, Scars, Vacuum massage, Dermal thickness, Dermal density

## Abstract

**Background:**

Vacuum massage is a non-invasive mechanical massage technique invented to treat burns and scars. To date, no effects of vacuum massage on thickness and density of human scar tissue have been reported. The process in which external stimuli are converted into biochemical responses in the cell is known as mechanotransduction. In the skin endothelial cells, fibroblasts and myofibroblasts embedded in the extracellular matrix (ECM) sense mechanical stimuli (created by vacuum massage) and may promote intracellular processes leading to matrix remodelling. Since mechanotransduction could be a plausible working mechanism for vacuum massage as an anti-scarring therapy, this study aims to investigate the short-term effects of vacuum massage on thickness and density of epidermis and dermis in burn scars in order to find proof of ECM remodelling.

**Methods:**

A one group experimental study was performed. Patients with burn scars on upper extremities, lower extremities, and trunk were recruited for participation in this study. The DUB®cutis 22 MHz ultrasound scanner was used to assess thickness and density of the epidermal and dermal skin layers. After baseline measurements, vacuum massage was performed according to a pre-defined protocol. Measurements were carried out at 5 min, 30 min, 1 h, and 2 h post-intervention.

**Results:**

Thirteen scar sites from 9 different patients were investigated. In 8 out of the 13 scar sites, a disruption of the epidermis was noticed after the vacuum massage. Five minutes after the intervention, epidermal density decreased statistically significantly (*p* = .022) and dermal thickness increased (*p* = .018). Both changes lasted for more than 1 h, but after 2 h, the changes were no longer statistically significant. Dermal density decreased significantly (*p* = .048) immediately after the intervention, and this decrease was still present after 2 h (*p* = .011).

**Conclusions:**

Preliminary results show that the disruption of the epidermis may indicate that vacuum massage could be able to actually breach the skin barrier. The statistically significant changes in the dermal layers could suggest an increased ECM production after vacuum massage.

## Background

In the burn population, hypertrophic scarring occurs in 67 % of the cases [1] and often leads to long-term impairment and disability [2]. Hypertrophic scars contain an overload of primarily type III collagen oriented parallel to the epidermal surface with multiple nodules containing myofibroblasts, large extracellular collagen filaments and abundant acidic mucopolysaccharides [3]. An overproduction of fibronectin and other fibroblast proteins is demonstrated suggesting either pathological persistence of wound healing signals or a failure to downregulate wound-healing cells [4]. It is generally accepted that hypertrophy and scar contraction can be minimised by reducing mechanical tension [5]. In the skin and other connective tissue, the process in which external mechanical stimuli are converted into biochemical responses inside the cell is known as mechanotransduction [6].

In the skin adherent cells including endothelial cells, fibroblasts and myofibroblasts embedded in the extracellular matrix (ECM) (or cellular substrate) sense tension (e.g. tension, shear and compression forces) originating from the environment [7]. Tension is transmitted as chemical signals via ECM contacts, leading to reorganisation of the cytoskeleton and the creation of specific signals that modulate gene expression (in the nucleus). Once the cell nucleus receives the appropriate signals, normal cellular processes are engaged. To sum up, the mechanical stimulus on the outside of the cell promotes intracellular processes leading to matrix remodelling [6]. The ECM is the largest component in normal skin; it plays a crucial role in the different wound healing processes [8]. After wound closure (after the inflammation and proliferation phase), the immature scar starts the remodelling phase; the ECM molecules, which are disorganised, are realigned and cross linked. Abnormal ECM reconstruction, particularly abnormal collagen remodelling, during wound healing leads to the formation of hypertrophic scars. In normal scars, small parallel bundles of collagen are present with skin appearing flat and discoloured while in hypertrophic scars, thin collagen fibres with increased synthesis and crosslinks result in raised scars [9]. The dermal orientation of the fibrous matrix differs from normal tissue [10], and there exists larger collagen density and larger fibre size in scars compared to normal tissue [9]. Hence, the characteristics of scars are a result of altered structure and composition in the dermis and the most important difference with normal skin tissue lies in the orientation of the fibrous matrix [8].

Mechanotherapy or the clinical application of mechanotransduction is the employment of mechanical means for the cure of disease [11]. It has had several definitions and involves the physical therapy (therapeutic exercise, massage therapy and orthopaedic rehabilitation) prescribed to promote the repair and remodelling of injured tissue. It is suggested that physical therapy also helps in healing or homeostasis of tissue outside the musculoskeletal system and may be able to oppose against specific pathophysiology and diseases [11]. Recently, new insights in the working mechanism of physical therapy have emerged and have led to a new definition for mechanotherapy: therapeutic interventions that reduce and reverse injury to damaged tissue or promote the homeostasis of healthy tissue by mechanical means at the molecular, cellular or tissue level [11].

Physical therapy modalities for scar treatments involve amongst other massage therapy as best practice [12], which can be manual or mechanical. One of these mechanical massage techniques is vacuum massage, also known as depressomassage [13], vacuotherapy [14] or Endermologie® [15]. Vacuum massage lifts the skin by means of suction, creates a skin fold and mobilises that skin fold [15–17]. In the late 1970s, this therapy was introduced to treat traumatic or burn scars [18]. Although vacuum massage was invented to treat burns and scars, one can find very few literature on the effects of this intervention. The effectiveness of vacuum massage has however not been widely proven. This study is part of a larger study in which the clinical effectiveness of vacuum massage is examined (a manuscript on the long-term effects of vacuum massage on clinical parameters is in preparation for submission). However, we found the underlying mechanisms of vacuum massage and mechanotherapy very important to give insight into what occurs in the scarred skin after mechanical massage. Massage therapy influences dysfunctioning of a burn patient in several domains of the International Classification of Functioning, Disability and Health (ICF). The ICF was created by the World Health Organisation and is a framework that describes the impact of a condition and incorporates physical, emotional, environmental and social aspects of daily functioning [19]. The domains of the ICF describes impairments in body structures and body functioning, activity limitations and participation restrictions. Effects of massage therapy on impairments in body functions (e.g. on pain and itch [20–22]) have been demonstrated in the burn population, but we found it important to know what influence is seen on body structures (actual change in the human skin).

Since mechanotransduction due to mechanical forces such as shear tension or compression could lead to collagen alterations and collagen re-orientation; this could be a plausible working mechanism for vacuum massage as an anti-scarring therapy. Significant changes in dermal thickness and density assessed by ultrasonography may be related to oedema and increased ECM production [23]. Results on animal models showed that as a result of vacuum massage, collagen content increased to as high as 130 % in long term treatments [16]. Effects were found on collagen alteration of fibroblasts phenotype and collagen orientation (more longitudinal). Results were dependent on the operator using the device, and the results were proportional to the number of treatments.

The goal of this study was to assess changes in the scar at a structural level (ICF domain body structures) in a non-invasive way. Thickness together with height and depth of the scars can be measured with ultrasonography [24]. The change in scar thickness measured with ultrasound has been used to assess the maturation of hypertrophic scars [25, 26] and can be used to compare hypertrophic scar thickness between patients [26] and evaluate intervention outcomes [25]. Ultrasonography can be used to examine therapeutic strategies on healing scars [27]; in dermatology, high resolution B scan ultrasound has enabled non-invasive assessment of different skin pathologies and has provided morphologic information of skin structures [28].

This study seeks to prove the earlier suggested effects and the remodelling of the ECM [6] by evaluating the short-term effects of vacuum massage on epidermal and dermal thickness and density in scarred skin measured with a high resolution B scan ultrasound.

## Methods

### Ultrasound measurement

With ultrasonography, high-frequency sound pulses are beamed into the skin and reflected at structural interfaces within tissues, where high acoustic impedance gradients are encountered [28]. For this study, thickness of the scars was determined by high-frequency ultrasound 22 MHz (DUB®Cutis, Taberna pro medicum, Lueneburg, Germany) and expressed in micrometres. Using this 22 MHz transducer, it was possible to visualise structures up to approximately 8 mm in depth. Sound is coupled from the transducer to the tissue by water in the scanning head of the probe to provide minimal attenuation of the ultrasound signal [29].

The probe was placed perpendicularly on the skin and a live feed displayed an area capable of measurement. Once completed, a snapshot of the ultrasound output was saved in the software on a computer. The A-scan image revealed the amplitude of the signals reflected by the borders of the different skin layers. The thickness of the dermis was measured by identifying the epidermis-dermis and dermis-subcutis interfaces [30] and was calculated by dividing the velocity of the ultrasound signal in the skin (approx. 1600 ms^−1^) by the transmission time of the signal [31]. The two-dimensional B-scan was used to calculate densitometric values (or density) of each lesion using pixel density converted into a 256-colour scale [32]. The region of interest was determined, and the values for epidermal and dermal thickness and density were registered. The ultrasound examination of the scars demonstrates the architecture of the skin. There is an entry echo line representing the gel-water-stratum corneum echo signals from the epidermis; the next change in echogenicity is the interface with the dermis and the start of the deep echo-lucent (black) area is the interface between dermis and subcutaneous fat [33]. The region of interest for which the values were to be determined was delineated by hand. The test site was marked with a surgical pencil, drawing the boundaries of the probe head (placed perpendicularly to the skin) and taking a picture to assure body position and exact location of the probe for the repeated measurements. The assessor in this study was a physical therapist trained in ultrasound probe application. The treatment was always performed by the same physical therapist specialised in vacuum massage therapy.

### Participants

Patients were recruited in Oscare, organisation for burns, scar aftercare and research in Antwerp, Belgium. Only patients with an age between 18 and 70 years qualified for the present study. Patients with Caucasian skin type and burn scars located on upper extremities, lower extremities and trunk (excluding the sole of the feet and hand palms) were included because of accessibility of the measure probe. Women in their last 3 months of pregnancy, patients with extremely high sensitivity for skin irritations, central neurological conditions, peripheral paralysis and diabetes were excluded for selection. Furthermore, patients who were obliged to take one of the following medications: Aspirin, Warfarin, Marcumar, Methrotrexate and Cyclosporin were excluded.

### Assessment and intervention protocol

**Fig. 1:**
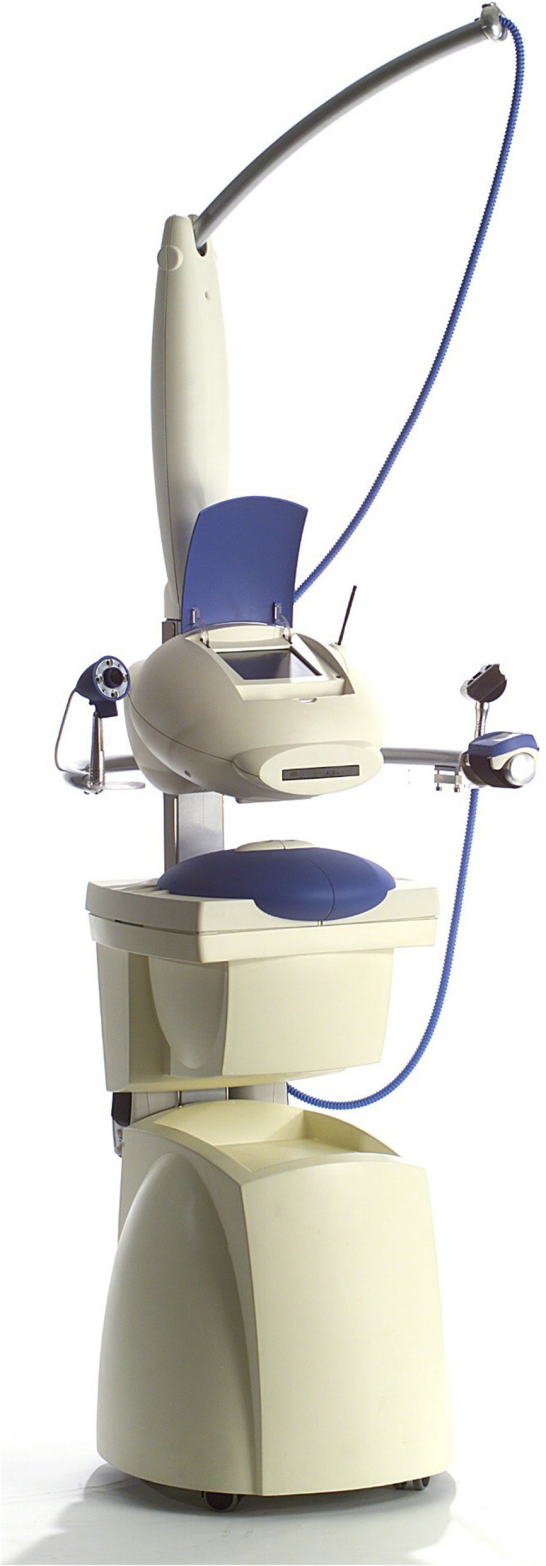
The PRUS vacuum massage device

**Fig. 2:**
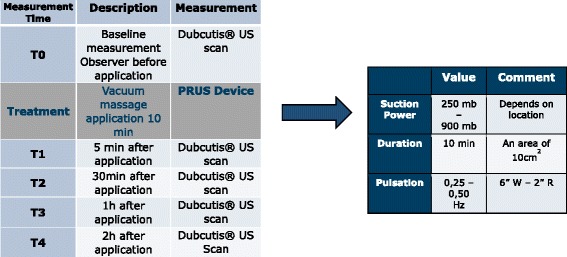
Assessment protocol with inclusion of the treatment protocol of the vacuum massage. *US* ultrasound, *mb* millibar, *Hz* Hertz, *W* working time, *R* resting time, *”* seconds

Informed written consent was obtained from all patients. The assessment in this study started with a single observer scanning a marked (exact location of the probe and photographed) area of the scarred skin with the DUB®Cutis ultrasound followed by a single treatment with the PRUS device (F care systems NV, Antwerp, Belgium), subsequently followed by repeated ultrasound scanning at different time points. The application with the PRUS vacuum massage, which is shown in Fig. 1, was performed for 10 min covering an area of 10 cm^2^ (the exact protocols are presented in Fig. 2). The study protocol was approved by the ethics committee of the Hospital Network Antwerp (ZNA), Belgium (Ethical committee 009OG031, study number EC4549).

### Statistics

All data was analysed using SPSS 20 software package for Windows. The normality was analysed using the Kolmogorov-Smirnov test. The data followed normal distribution; hence, parametric tests were performed. Descriptive statistics were generated, and paired sample *t* tests with estimates of effect size were calculated to determine whether there was a statistically significant mean change in the epidermal and dermal thickness and density between the time points. We calculated effects size as Cohen *d*, with *d* defined as the difference between the 2 means divided by the pooled SD for those means. A *d* value of 0.20 is described as small, 0.50 as moderate and 0.80 as large [34]. Significance was set at 0.05. The bar charts in Figs. 4, 5 and 6 display the mean and the standard deviations.

## Results

**Fig. 3:**
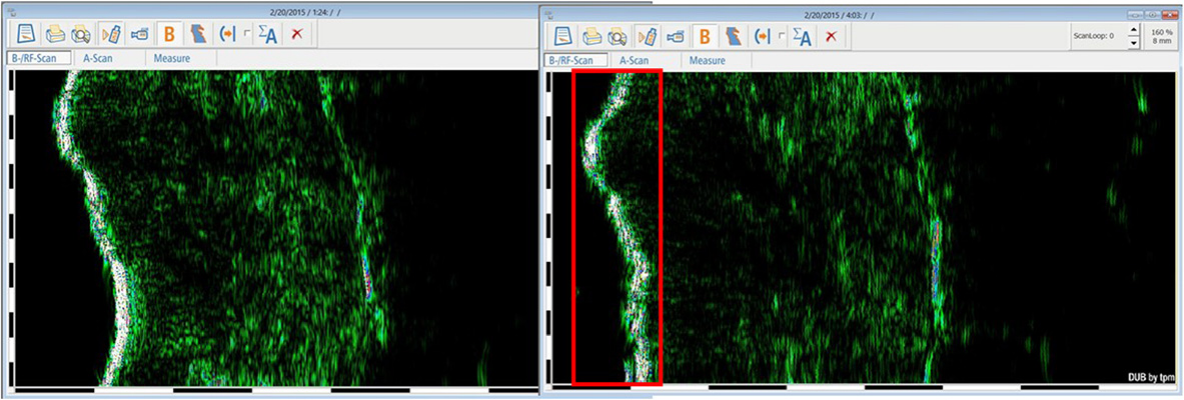
Illustration of disruption of the epidermis visible on the DUB®Cutis dermascan B image

This preliminary study consisted of 13 post-burn scar sites from nine different patients, three women and six men. The mean age was 24.56 ± 13.26 years, mean scar age was 16.26 ± 14.27 months and scars were located on the upper extremities (*n* = 3) and lower extremities (*n* = 7) and on the trunk (*n* = 3). As presented in Fig. 3, we found a disruption (visual presence of multiple echo lucent areas) of the epidermis in 8 out of 13 scar sites after the vacuum massage.

**Fig. 4:**
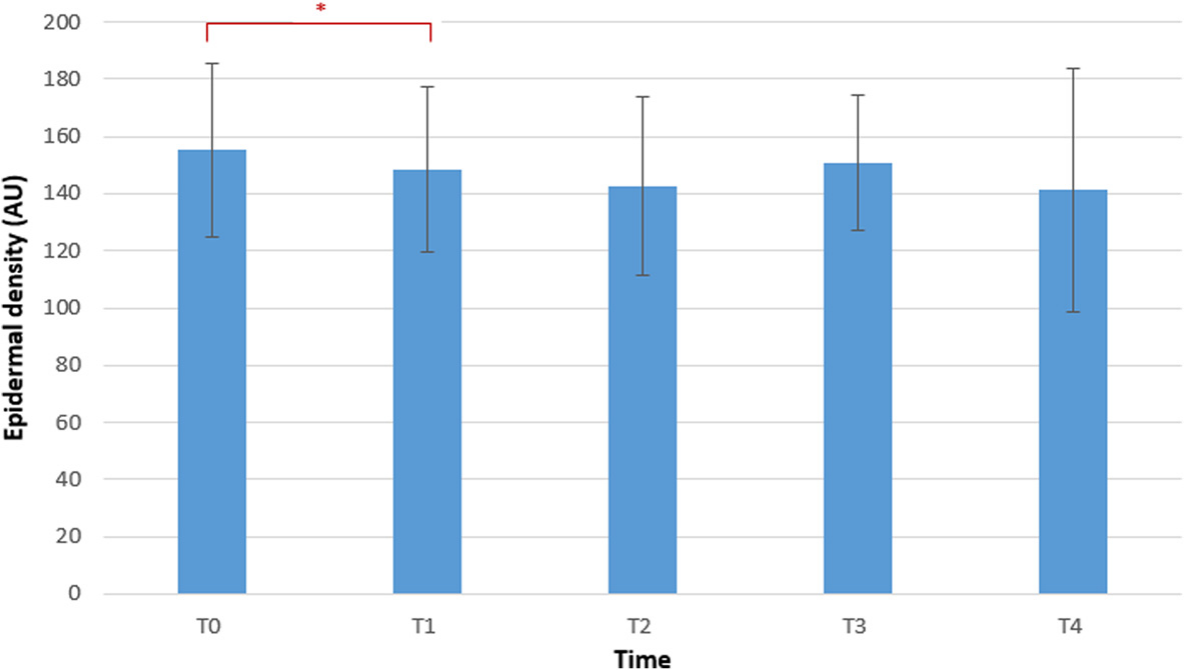
Bar chart showing the data distribution summaries of the epidermal density at the different time points. *p < 0.05. Bar chart showing the mean and the *error bar* represents the standard deviations. *T0* baseline measurement, *T1* 5 min after application, *T2* 30 min after application, *T3* 1 h after application, *T4* 2 h after application, *AU* arbitrary units

No significant changes were found in the epidermal thickness. Immediately after the intervention (T1), the epidermal density decreased statistically significant (*p* = 0.022) with a moderate effect size of −0.73. Two hours after the intervention (T4), the epidermal density did not show any significant changes (*p* = 0.096) compared to baseline and the effect size decreased to −0.61. This is illustrated in Fig. 4.

**Fig. 5:**
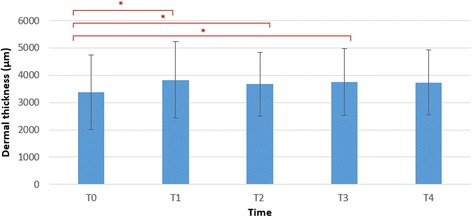
Bar chart showing the data distribution summaries of the dermal thickness at the different time points. *p < 0.05. Bar chart showing the mean and the error bar represents the standard deviations. *T0* baseline measurement, *T1* 5 min after application, *T2* 30 min after application, *T3* 1 h after application, *T4* 2 h after application, *μm* micrometre

Immediately after the intervention, the dermal thickness increased statistically significant (*p* = 0.018) with a moderate to large effect size of 0.76. After half an hour, the thickness decreased compared to the previous measurement but was still statistically significant increased when compared to baseline measurement (*p* = 0.046). After 1 h, we still observed a significant increase compared to the baseline measurement (*p* = 0.013). Although a decrease was observed 2 h after the intervention, the dermal thickness did not show any significant changes (*p* = 0.06) compared to baseline and the effect size decreased to 0.57 (Fig. 5).

**Fig. 6:**
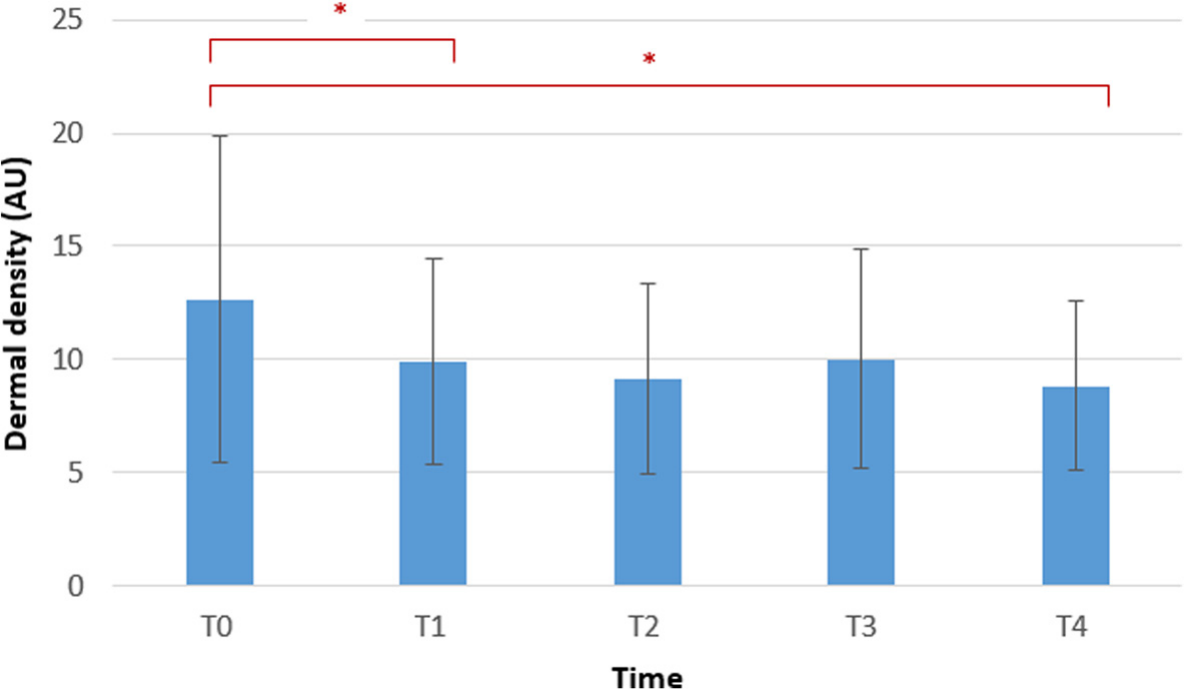
Bar chart showing the data distribution summaries of the dermal density at the different time points. *p < 0.05. Bar chart showing the mean and the error bar represents the standard deviations. *T0* baseline measurement, *T1* 5 min after application, *T2* 30 min after application, *T3* 1 h after application, *T4* 2 h after application, *AU* arbitrary units

Immediately after the intervention, the dermal density decreased statistically significant (*p* = 0.048) with a moderate effect size of −0.61. Two hours after the intervention, the dermal density was still decreased statistically significant (*p* = 0.011) with a large effect size of −0.83 (Fig. 6).

**Fig. 7:**
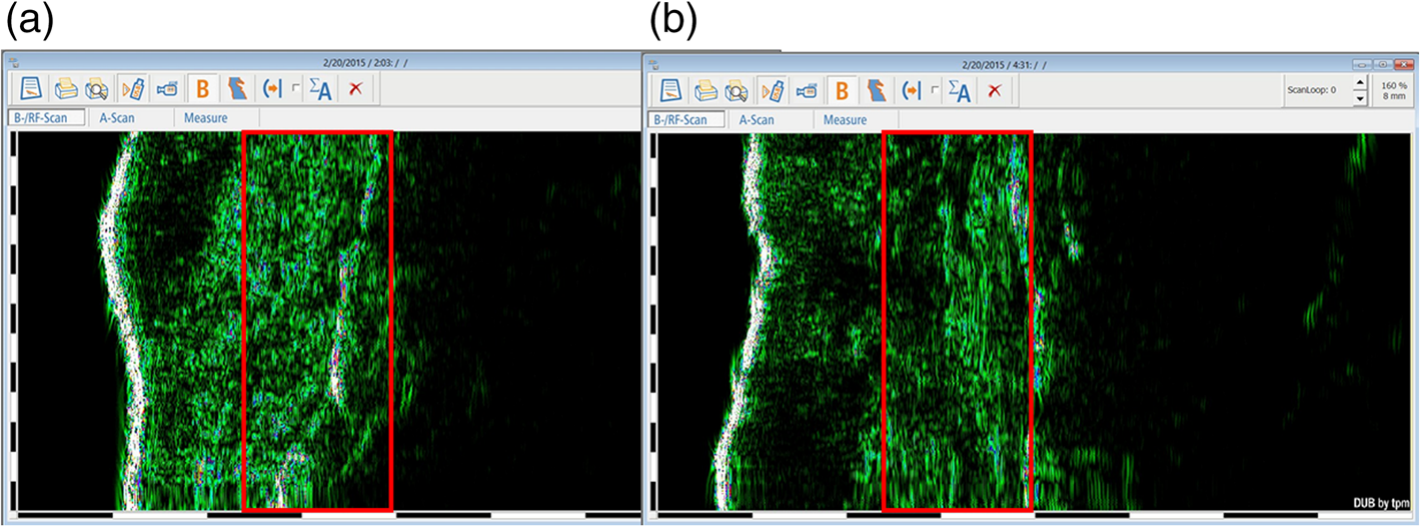
Illustration of dermal arrangement visible on the DUB®Cutis dermascan B image. **a** Prior to treatment with more nodular arrangement. **b** Post-treatment with more longitudinal arrangement

The echo images prior to treatments revealed nodular arrangement in the dermis. As illustrated in Fig. 7, a more longitudinal arrangement was observed after vacuum massage treatment.

## Discussion

This study investigated the short-term effects of vacuum massage on physical scar properties and found statistically significant changes in the epidermal density immediately after the intervention. Dermal thickness increased significantly immediately after the intervention; after 1 h, the dermal thickness decreased significantly compared to baseline and a trend was seen at 2 h compared to baseline. Dermal density decreased significantly after the vacuum massage treatment, and even at 2 h post-treatment, this decrease was still significant compared to baseline.

### Interpretation of the findings

These findings are in line with the findings of Hesselstrand et al. (2008) who also found an increase in dermal thickness and reduced echogenicity in patients with scleroderma which was explained by oedema formation and an increased ECM production [23]. The disruption of the epidermis (assessed by the visual presence of multiple echo lucent areas) and the decrease of the epidermal density might indicate that the intervention could be able to actually breach the skin barrier [17].

The initial pre-intervention echo images revealed a nodular arrangement in the dermis; after the intervention, the dermal fibre arrangement was more longitudinal and wave-like patterned. This may indicate collagen realignment due to the vacuum massage. For dermal density, the effects even last until 2 h after the intervention, which could indicate that ECM restructuring still goes on. These results are in line with the findings of Adcock et al. who reported a reorganisation of the ECM with increasing collagen synthesis and realignment of the collagen fibres after vacuum massage on Yorkshire pigs [17], both signs of ECM restructuring. We hypothesised that vacuum massage may be a potential anti-scarring therapy leading to collagen re-orientation drawn on the underlying concept of mechanotransduction. The observed collagen arrangement in the current study needs further data research with histological examination to establish these dermal changes, and further exploration of mechanotransduction pathways in vacuum massage is needed. Somewhat similar to our findings, a recent study on the effect of pressure therapy in post-burn hypertrophic scars revealed reduced dermal cell density and altered collagen fibre arrangement in histological examination [35]. Myofibroblasts responsible for pathological scar formation in hypertrophic scars were abundantly present in the pre-treated scars; the applied mechanical pressure therapy significantly suppressed myofibroblast activity after which the myofibroblasts disappeared by apoptosis leading to improved collagen fibre alignment [35]. Another recent study reviewed the role of mechanical forces at the molecular and tissue level in various physical therapy treatments (e.g. massage and shockwave therapy) [11]. The researchers emphasised the beneficiary role of vacuum-assisted closure (VAC) or negative pressure wound therapy (NPWT) as a ‘mechanotherapy’. Since their mechanism is similar to that of vacuum massage, a similar beneficiary role may be expected.

Efficacy of massage has been described in prior research to stimulate cell signalling pathways and activate potentially immunomodulatory pathways [36]. Massage reduces cellular infiltration, inflammation and edema in muscles [37]. This study, although preliminary, shows positive effects of mechanical massage on the physical properties of scarred skin. Mechanotherapy together with mechanotransduction is a promising field that may promote healing of tendon, muscle cartilage bone [6] and also scarred skin [11]. Only the tip of the iceberg has been unravelled, and there is abundant room for further research in determining the exact effects of massage and vacuum massage on post-burn skin physical structures.

### Methodological considerations

There are several weaknesses in this study that should be addressed in future studies. Scar sites were located on the upper and lower extremities and the trunk in this study, and literature on normal skin has shown that there are substantial differences between skin thickness of the upper extremity and the lower extremity [38]. To reach a statistical power of at least 0.80, the required sample size for this investigation should be at least 17 patients. The average age of the scars was rather high and had a large SD; therefore; the results of this investigation cannot be generalised. The echogenicity of the skin depends on the dermal water content, on the amount of collagen and on the skin configuration [28]. The echo poor images recorded after the vacuum massage treatment may indicate a change of the collagen structure but can also be the result of an increase in water-binding properties or increased dermal water content [28]. Various high-frequency ultrasound instruments exist to evaluate scar thickness varying in costs, size and applicability. Although other authors demonstrated good inter- and intra-reliability for high-frequency ultrasound testing in post-burn scars [27], only recently, the DUB®Cutis ultrasound scanner was found reliable for dermal thickness and density for repeated measures by one or two different observers [39] in post-burn scars. Moreover, the instrument was found reliable to assess epidermal thickness for repeated measures by one observer. Comparing echo images with histological examination and this within a RCT design would be the next step to establish the current epidermal and dermal findings as a result of vacuum massage treatment. The short-term effects of vacuum massage were chosen to minimise bias by other treatments (pressure garments, hydration, silicone, physiotherapy). In this short time period, the patients remained in the centre (in the same room and if possible in the same position); this facilitated relocations.

Some authors describe ultrasonography as complicated and requiring professional training [40]; we agree that the probe application acquires thorough training since pressure influences the results of the epidermal layer. Relocation of the measurement site must be done under strictly standardised conditions. Moreover, we recommend to interpret a series of scans from the same patients together to enhance exact relocation of the measurement site. Despite the limitations, this study has some strengths. This study is the first to describe and hypothesise mechanotransduction as a possible working mechanism of vacuum massage on post-burn scars.

## Conclusions

In conclusion, the disruption of the epidermis might indicate that the effect of vacuum massage could be able to actually breach the skin barrier. The statistically significant changes in the dermal layers suggest oedema formation and an increased ECM production which could be attributed to an immediate mechanotransduction effect of vacuum massage on the remodelling of the ECM. Further research is needed to elucidate the preliminary findings of this study and the effects of different forms of mechanotherapy on the physical scar properties and beyond in patients with post-burn scars.

## Abbreviations

ECM, extracellular matrix; ICF, International Classification of Functioning, Disability and Health; NPWT, negative pressure wound therapy; VAC, vacuum-assisted closure ; ZNA, Hospital Network Antwerp
